# Development of a risk scoring system for prognostication in HIV-related toxoplasma encephalitis

**DOI:** 10.1186/s12879-020-05651-x

**Published:** 2020-12-04

**Authors:** Yao Li, Yan-Ming Zeng, Min Liu, Yan-Qiu Lu, Xue-Yan Liu, Yu-Lin Zhang, Zhong-Sheng Jiang, Tong-Tong Yang, Yan Sun, Ke Lan, Yao-Kai Chen

**Affiliations:** 1 Division of Infectious Diseases, Chongqing Public Health Medical Center, 109 Baoyu Road, Shapingba, Chongqing, China; 2 Department of medical imaging, Chongqing Public Health Medical Center, Chongqing, China; 3 Division of Infectious Diseases, Beijing Youan Hospital, Capital Medical University, Beijing, China; 4 Department of Infectious Diseases, Liuzhou People’s Hospital, Liuzhou, Guangxi China; 5 Division of Infectious Disease, Chengdu Public Health Clinical Medical Center, Chengdu, Sichuan China; 6 Department of Infectious Diseases, The Sixth People’s Hospital of Zhengzhou, Zhengzhou, Henan China; 7 Division of Infectious Disease, Longtan Hospital of Guangxi Zhuang Autonomous Region, Liuzhou, Guangxi China

**Keywords:** HIV, Toxoplasma encephalitis, Risk scoring system, Death, Retrospective study

## Abstract

**Background:**

This study aims to evaluate specific risk factors influencing prognosis of HIV-infected patients with toxoplasma encephalitis (TE) in order to develop a prognostic risk scoring system for them.

**Methods:**

This is a six-center retrospective study of hospitalized HIV/TE patients. Data including six-week mortality after diagnosis, baseline characteristics, clinical features, laboratory tests and radiological characteristics of eligible patients were assimilated for risk model establishing.

**Results:**

In this study, the six-week mortality among 94 retrospective cases was 11.7% (11/94). Seven specific risk factors, viz. time from symptom onset to presentation, fever, dizziness, CD4+ T-cell counts, memory deficits, patchy brain lesions, and disorders of consciousness were calculated to be statistically associated with mortality. A criterion value of ‘9’ was selected as the optimal cut-off value of the established model. The AUC of the ROC curve of this scoring model was 0.976 (*p* < 0.001). The sensitivity and specificity of the risk scoring model was 100.0 and 86.9%, respectively, which were 81.8 and 94.1% of this scoring model in the verification cohort, respectively.

**Conclusions:**

The developed scoring system was established with simple risk factors, which also allows expeditious implementation of accurate prognostication, and appropriate therapeutic interventions in HIV-infected patients with TE.

**Supplementary Information:**

The online version contains supplementary material available at 10.1186/s12879-020-05651-x.

## Background

Toxoplasmosis is a zoonotic parasitic disease caused by *Toxoplasma gondii* infection [1]. In HIV-infected patients, seropositivity for Toxoplasma antibodies is as high as 10–40%, and it is estimated that a third of those patients will eventually progress to toxoplasma encephalitis (TE) [2], particularly in those with CD4+ T-cell counts <50 cells/μL [3]. Unfortunately, the poor prognosis of TE generally leads to death or disability in HIV-infected patients. Even after anti-toxoplasma treatment, the mortality of HIV-associated TE during hospitalization is still as high as 29.9% [4]. Thus, early identification of high-risk cases at the diagnosis of TE is crucial for improving prognosis and reducing TE-associated mortality among HIV-infected patients.

Previous studies have reported on potential predictors of poor prognosis of central nervous system toxoplasmosis. For example, independent risk factors for death among HIV-infected patients with TE were acute kidney injury (AKI) and hyponatremia [5]. CD4+ T-cell counts and Glasgow Coma Scale (GCS) scores were independently associated with poor outcome (modified Rankin Scale > 2) in HIV-infected patients with severe cerebral toxoplasmosis [6]. It is therefore apparent that there have been studies considering factors related to the risk of poor prognosis in TE, and that the reported factors are not specific, and are mainly restricted to specific laboratory tests. In addition, there is at present no risk stratification scoring model for TE among HIV-infected patients specifically designed to prognosticate outcomes. Therefore, the development of a user-friendly scoring model with clear outcome prognostication benefit, and a favorable predictive value, is urgently needed.

In this study, risk factors for death were analyzed using the data from 94 HIV-infected patients with TE. This retrospective cohort study aimed to determine the risk factors related to prognosis, and establish a simple scoring system based on baseline characteristics, clinical features, laboratory tests, and radiological characteristics, and subsequently to identify its effectiveness in a prospective validation cohort of HIV-infected patients with TE.

## Methods

### Patients

This was a six-center retrospective study involving hospitalized patients. One hundred fifty-six HIV-infected patients admitted to hospital from May 2013 to September 2019, and diagnosed with TE were eligible to participate in the present study. Six-week mortality after diagnosis was defined as the study outcome. Six-week is the time point at the end of anti-Toxoplasma therapy recommended by the National Institutes of Health (NIH) guidelines [7], and is a vital time point to assess the efficacy of the treatment, and adverse outcomes such as mortality [8]. Sixty-two patients were excluded from the retrospective cohort, due to the absence of outcome data, demographic characteristics, clinical features, laboratory test results, or radiological findings. Subsequently, the demographic and clinical data of 94 patients were used for the development of our scoring model. Forty-five patients who were admitted to hospital between October 2019 and March 2020 and with appropriate and available data were eligible to be included in our validation cohort.

### Standard protocol approvals, registrations, and patient consents

The present study was approved by the institutional review board of Chongqing Public Health Medical Center (No. 2019–003-02-KY). The institutional review board waived the requirement for written informed consent, since the present study was retrospective and all patients’ data were analyzed in anonymity.

The raw data may be requested from the first author and corresponding author, with administrative permissions of the review board of the Chongqing Public Health Medical Center, first author and corresponding author.

### Data collection

Six-week mortality after diagnosis, details of baseline characteristics, clinical features, laboratory tests, and radiological characteristics of 94 patients were collected, including: gender, age, ART initiation, time from symptom onset to presentation, headache, dizziness, fever, vomiting, memory deficits, cognitive impairment, disorders of consciousness, dysphasia, meningeal irritation signs, CD4+ T-cell counts, patchy CT/MRI lesions, and ring-enhancing CT/MRI lesions.

### Statistical analysis

All data analysis was executed using the Statistical Package for the Social Sciences (SPSS) Version 23.0 software (IBM-SPSS, Armonk, NY, USA) and MedCalc Version 18.9 (MedCalc Software Ltd., Ostend, Belgium). Univariate logistic regression analysis was performed to assess potential predictors of non-survival, and potential factors with *p*-values < 0.20 were included in the multivariate stepwise forward logistic regression analysis for score assignment. A receiver operating characteristic (ROC) curve of this scoring model was then generated, and the area under the curve (AUC) of ROC curve was calculated to assess model accuracy. Also, a cut-off value with optimal sensitivity and specificity was identified in the validation cohort.

## Results

### Patients

The six-week mortality among 94 retrospective cases in the present study was 10.6% (10/94). As shown in Table 1, 38.3% (36/94) of patients were aged ≥40 years old, 74.5% (70/94) were male, around one thirds of the cohort had headaches (32/94), dizziness (31/94), or fever (32/94), and 25.5% (24/94) of cases had vomiting as a symptom at baseline. The study flow diagram is displayed in the supplementary information Figure 1.

**Table 1:** Univariate logistical regression analysis

Variable	Total	Death	Survival	***p***	OR (95%CI)
Age (years old):	94			0.570	1.510 (0.364, 6.256)
< 40;		7;	51;		
≥ 40		3	33		
Gender:	94			0.259	3.393 (0.407, 28.297)
Male;		9;	61;		
Female		1	23		
Fever:	94			0.123	0.109 (0.636, 43.550)
Yes;		1;	31;		
No		9	53		
Dizziness:	94			0.136	0.500 (0.604, 41.392)
Yes;		1;	30;		
No		9	54		
Headache:	94			0.776	1.230 (0.296, 5.117)
No;		3;	29;		
Yes		7	55		
Vomiting:	94			0.732	1.286 (0.305, 5.426)
No;		7;	63;		
Yes		3	21		
Memory deficits:	94			0.030	4.500 (1.157, 17.500)
No;		4;	63;		
Yes		6	21		
Cognitive impairment:	94			0.133	3.950 (0.657, 23.749)
No;		8;	79;		
Yes		2	5		
Disorders of consciousness:	94			0.005	7.400 (1.817, 30.144)
No;		5;	74;		
Yes		5	10		
Dysphasia:	94			0.008	6.375 (1.608, 25.274)
No;		4;	68;		
Yes		6	16		
Meningeal irritation signs:	94			0.053	6.750 (0.979, 46.551)
No;		8;	81;		
Yes		2	3		
Time from symptom onset to presentation:	94			0.193	2.567 (0.621, 10.604)
< 15 days;		3;	44;		
≥15 days		7	40		
ART initiation:	94			0.421	1.731 (0.455, 6.582)
No;		4;	45;		
Yes		6	39		
CD4+ T-cell counts:	94			0.005	7.982 (1.880, 33.890)
≥ 25 cells/μL;		3;	65;		
< 25 cells/μL		7	19		
Patchy lesions on MRI:	94			0.192	2.437 (0.639, 9.305)
No;		4;	52;		
Yes		6	32		
Ring-enhancing lesions on MRI:	94			0.790	0.833 (0.218, 3.188)
No;		4;	30;		
Yes		6	54		

### Univariate logistic regression analysis

Sixteen variables, considered to be associated with non-survival in HIV-associated TE cases, were selected for univariate logistic regression analysis. The post-analysis results revealed that significant statistical differences existed among certain variables, viz. memory deficits, disorders of consciousness, dysphasia, and CD4+ T-cell counts between survivors and non-survivors (*p* < 0.05). Details were illustrated in Table 1.

### Multivariate logistic regression analysis and scoring of variables

Ten variables had a *p*-value < 0.2 from the results of univariate logistic regression analysis, and all of them were selected for multivariate logistic regression analysis. Finally, seven variables were retained in the optimal regression equation with *p* < 0.05 through forward logistic regression stepwise regression analysis, and further incorporated for score assignment. Patchy brain lesions present on CT or MRI was the variable with the lowest regression coefficient (2.006), which means that “patchy brain lesions” as a risk factor, has the lowest influence on death, and was thus assigned the lowest arbitrary score of one. The remaining six variables were as follows: time from symptom onset to presentation, fever, dizziness, CD4+ T-cell counts, memory deficits and disorders of consciousness, and which were assigned a score in sequence, after dividing each regression coefficient of six different risk factors by 2.006 to determine the score (in units of 0.5), as shown in Table 2.

**Table 2:** Multivariate logistic regression analysis and scoring variables

Variables	Coefficient	***p***	score
Fever	7.764	0.031	4
Dizziness	3.624	0.099	2
Memory deficits	3.066	0.07	2
Disorders of consciousness	5.065	0.033	3
Time from symptom onset to presentation ≥15 days	3.34	0.048	2
CD4+ T-cell counts < 25 cells/μL	5.431	0.011	3
Lesions manifesting as patchy	2.006	0.180	1

**Fig. 1:**
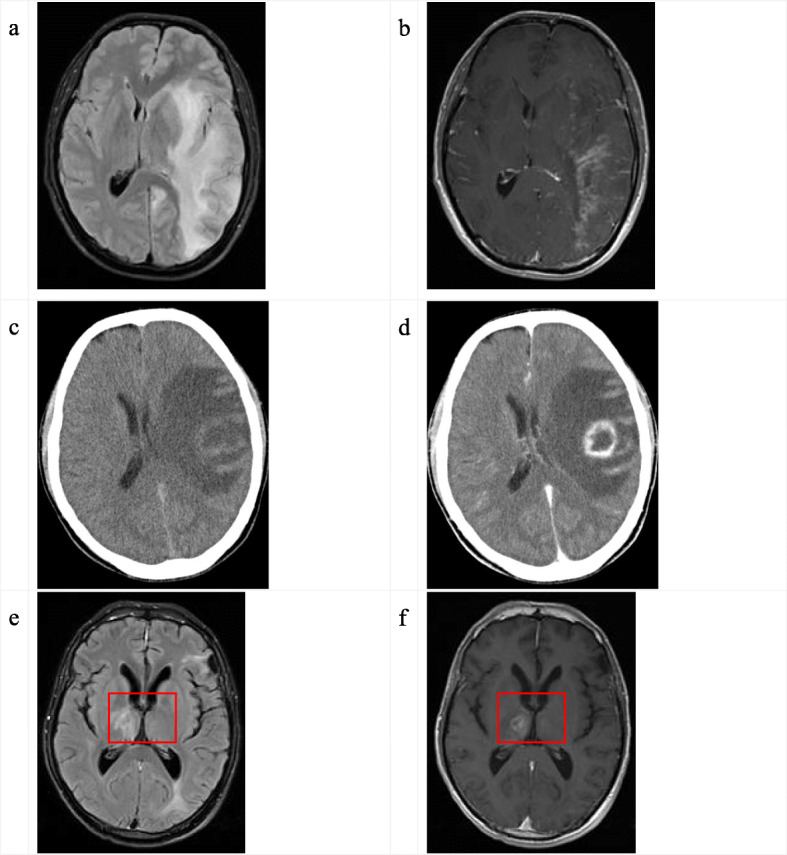
Radiological images of three cases of non-survival. The T2 flair series showed large patchy abnormal signals on the left frontal lobe, temporal lobe, and occipital lobe **(a)**. T1 gadolinium-enhanced scans showed multiple patchy enhancements in the left frontal lobe, temporal lobe, and occipital lobe **(b)**. A CT scan of the head revealed a large patch of low-density shadow on the right temporal lobe **(c)**, and an enhanced CT scan revealed a ring-shaped enhancement on the right temporal lobe **(d)**. The T2 flair series showed slice-like and patch-like abnormal signals at the junction of the right thalamus, left frontal lobe, and left temporal and occipital lobes **(e)**. A T1 gadolinium-enhanced scan showed a ring-shaped enhancement in the right thalamus **(f)**. Note: Lesions on temporal lobe were reported to be related to memory deficits (a-d); lesions on thalamus were reported to be related to disorders of consciousness (e-f).

TE cerebral lesions on temporal lobe structures of non-survivor cases were assumed to be related to memory deficits [9], as shown in Fig. 1 a-d, and typical patchy lesions in the thalamus of one non-survivor, which was considered to be associated with disorders of consciousness [10], as shown in Fig. 1(e-f), respectively.

### Score distribution and ROC curve of the scoring model

**Fig. 2:**
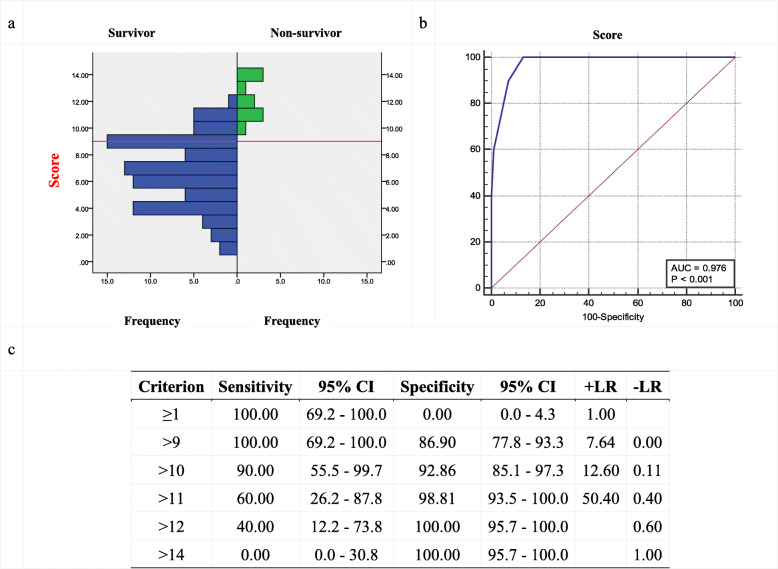
The development of the scoring model. **(a)** distribution of cases of survival and cases of non-survival in HIV-infected patients with TE; **(b)** ROC curve of the scoring model; **(c)** criterion values and coordinates of the ROC curve

The score distribution of survivor and non-survivor cases of HIV-infected patients with TE is shown in Fig. 2a. In conjunction with our established model, as shown in Fig. 2a, a criterion value of ‘9’ was selected as the optimal cut-off value. The AUC of the ROC curve of this scoring model was 0.976 (*p* < 0.001), and the sensitivity and specificity of this scoring model at the cut-off value of “9” was 100.0% (95%CI, 69.2–100.0%) and 86.9% (95%CI, 77.8–93.3%), respectively, as shown in Fig. 2b and c.

### Verification and accuracy of the scoring model

**Fig. 3:**
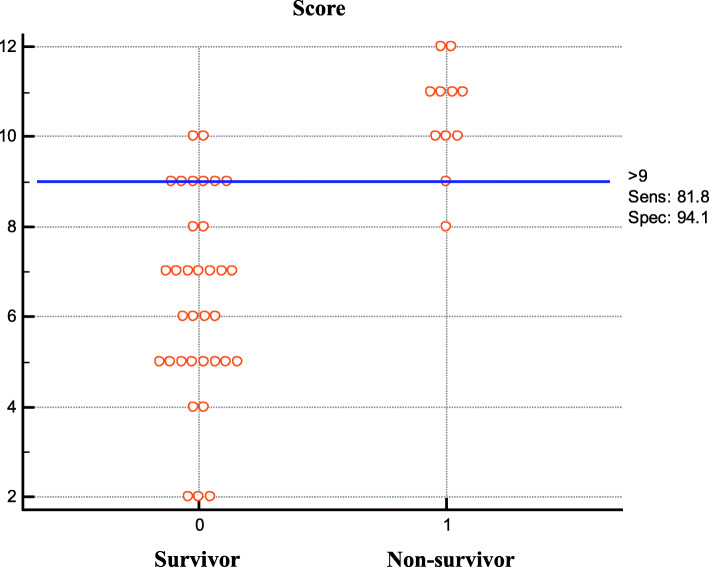
Integral distribution of HIV-infected TE patients in the validation cohort

Forty-five HIV-infected cases with TE were involved in the scoring model verification cohort, and the sensitivity and specificity of the model in this cohort was 81.8 and 94.1%, respectively. The accuracy of the scoring system in the verification cohort was 91.1% (41/45), as shown in Fig. 3.

## Discussion

The prevalence of co-infection with *Toxoplasma gondii* and HIV ranges from 25.1 to 60.7% in different countries [11]. The mortality of HIV-associated TE during hospitalization, with appropriate anti-toxoplasma treatment, may be up to 30% [4]. The 6-week mortality in the present study was 11.7%. In order to effectively reduce mortality in patients with HIV-associated TE, timely targeted intervention based on specific prognostic factors is a sensible undertaking. Thus, we conducted this study, and aimed to discover specific risk factors of 6-week mortality, and to establish a scoring system model to credibly predict poor outcomes in patients with HIV-associated TE.

Thus far, few studies have described the relationship between proposed risk factors and poor outcomes among HIV-infected patients with TE. Libório et al. conducted a retrospective cohort study and found that AKI (OR = 8.3) and hyponatremia (OR = 9.9) were independent risk factors for death in HIV-infected patients with TE [5]. Another retrospective cohort study regarding patients with severe cerebral toxoplasmosis conducted by Sonneville et al., showed that a CD4+ T-cell counts < 25 cells/μL (OR = 2.7), and a GCS score ≤ 8 (OR = 3.1) were independently associated with poor outcomes (modified Rankin Scale score ≥ 2) at 3 months [6]. Conversely, CD8+ T-cell counts were reported to play a dominant role as a protective factor in AIDS patients with chronic toxoplasmosis [12]. Hoffmann et al. found that overall survival of AIDS patients with toxoplasmosis was significantly improved in those patients diagnosed after 1996 (the highly-active antiretroviral therapy era), those without a previous AIDS-defining illness, those aged < 45 years, and those with a LDH level < 300 U/L [13].

Based on the results of these preceding studies and the integrity of the data in the present study, we selected 16 different potential risk factors as prognostic factors for screening and scoring model development. The results of univariate and multivariate logistic regression analysis revealed that fever and dizziness were protective factors in survivors of HIV-associated TE, suggesting that patients with these two encephalopathy-related symptoms are more likely to seek medical treatment expeditiously, thereby avoiding treatment delay and reducing mortality. In addition, the significant statistical difference in overall survival between the time from symptom onset to presentation ≥15 days and < 15 days (*p* = 0.048) in survivors and non-survivors, also reinforces the benefits of timeous treatment. We observed that CD4+ T-cell counts < 25 cells/μL was associated with non-survival at 6-weeks after diagnosis (OR = 7.982) in HIV-infected patients with TE. Memory deficits usually manifested as episodic and semantic learning disability, and was reported to be related to damage of medial temporal lobe structures, and the hippocampus [8]. Previous studies have confirmed that at a neuroanatomic level, activation of the cerebral cortex occurs with passage of sensory data from the upper brainstem via the reticulo-thalamo-cortical and extrathalamic pathways [10]. Thus, toxoplasmic disorders of consciousness may be related to lesions of the thalamus and its surrounding tissues [10]. Patchy cortical lesions are usually scattered, usually involving multiple areas of the brain, and are considered to be associated with non-survival in this study. The present developed scoring model is based on data extracted from 94 retrospective case files, and is correlated with high sensitivity and specificity. The scoring system has also performed well when tested in the validation cohort. Also, in this newly-developed scoring system, simple risk factors that are readily available from patient clinical records allows for the seamless and expeditious implementation of the scoring system, allowing prognostication and consequent appropriate therapeutic interventions to be implemented in an expeditious manner.

There are a few limitations to the present study. Firstly, as a consequence of the widespread use of ART in China, only modest numbers of cases with complete data were involved in the scoring model development, and also in the model verification cohort. Secondly, due to the absence of adequate data for some risk factors, screening for risk factors has introduced an inherent degree of bias to the present study. Thirdly, this scoring model is based on Chinese HIV-infected patients with TE. Whether the scoring system is applicable to TE patients without HIV, or to other population groups requires further research. Lastly, long-term mortality and neuropsychological patient outcomes cannot be extrapolated from our findings.

## Conclusions

The developed scoring system was established to evaluate specific risk factors influencing prognosis of HIV-infected patients with TE. The scoring system included seven risk factors, use of which allows expeditious application of accurate prognostication, and implementation of appropriate therapeutic interventions in HIV-infected patients with TE. However, whether the scoring system is applicable to TE patients without HIV, or to population groups other than Chinese, warrants further research.

## Supplementary Information

**Additional file 1: Supplementary Figure 1.** Study Flow Diagram.

## Data Availability

The raw data may be requested from the first author and corresponding author, with administrative permissions of the review board of the Chongqing Public Health Medical Center, first author and corresponding author. All patient data has been de-identified.

## Abbreviations

AIDS: Acquired immunodeficiency syndrome
TE: Toxoplasma encephalitis
HIV: Human immunodeficiency virus
AKI: Acute kidney injury
LDH: Lactate dehydrogenase
GCS: Glasgow coma scale
SPSS: Statistical Package for the Social Sciences
ROC: Receiver operating characteristic
AUC: Area under the curve
CT: Computed tomography
MRI: Magnetic resonance imaging
PCR: Polymerase chain reaction
ART: Antiretroviral therapy
