# Supplementary material for: Development of a risk scoring system for prognostication in HIV-related toxoplasma encephalitis
Source: BMC Infect Dis. 2020 Dec 4;20:923. doi: 10.1186/s12879-020-05651-x (PMC7716502; doi:10.1186/s12879-020-05651-x)
Supplement: Supplementary file 1 — Additional file 1: Supplementary Figure 1. Study Flow Diagram. [file 12879_2020_5651_MOESM1_ESM.doc]

**Supplementary figure 1.** Study Flow Diagram

**156** patients were screened

**62** patients removed due to the missing data

**94** patients were eligible

Survivor

(**n=84**)

Non-survivor

(**n=10**)

Univariate logistic regression analysis was performed to assess potential predictors

Potential predictors with *p*-values < 0.20 were included in the multivariate logistic regression analysis

Assigning a score for each variable

Verification of the scoring system with **45** cases

**Supplementary figure 1.** Study Flow Diagram
